# Improvement in Mechanical Pain and Disability in Patients With Flatfoot After the Use of Medical Shoes

**DOI:** 10.7759/cureus.80279

**Published:** 2025-03-09

**Authors:** Hail Turki Alharthi, Abdulaziz M Alraddadi, Abdullah M Alraddadi, Renad A Alshaer, Emad A Alsaedi, Abdulrahman M Alraddadi, Suduf A Alkuhayli

**Affiliations:** 1 Orthopedics, Taif University, Taif, SAU; 2 Medicine and Surgery, Al Rayan National College of Medicine, Madinah, SAU; 3 College of Medicine, Majmaah University, Majmaah, SAU; 4 Medicine, Taif University, Taif, SAU; 5 Internal Medicine, King Salman bin Abdulaziz Medical City, Madinah, SAU; 6 Medicine and Surgery, Ibn Sina National College, Jeddah, SAU

**Keywords:** disability, flat foot, footwear, mechanical pain, medical shoes, orthotic device, pes planus, saudi arabia

## Abstract

Background and objective

Flatfoot (pes planus), a common condition associated with pain and functional limitations, can significantly impact the quality of life. This study aimed to evaluate the effectiveness of medical shoes in reducing pain and improving functional mobility among individuals with flatfoot in Saudi Arabia.

Methods

A retrospective cohort study was conducted over one year involving 400 participants aged 18-65 years. Participants were categorized into two groups: those using medical shoes and those not using medical shoes. Data on pain levels, functional mobility, and disability were collected using validated scales such as the Foot Function Index (FFI). Statistical analyses included the Mann-Whitney U test and logistic regression to compare outcomes between groups.

Results

Participants using medical shoes reported significantly reduced pain levels, particularly in terms of standing (p=0.024), end-of-day pain (p=0.010), and worst pain severity (p=0.001). Improvements in functional mobility were also noted, with significant reductions in difficulty when walking four blocks (p=0.012), climbing stairs (p=0.014), and standing on toes (p=0.007). However, no significant differences were observed in long-term disability indicators, such as the use of assistive devices or physical activity restrictions (p>0.05). Logistic regression analysis revealed that medical shoe use was associated with significant pain and mobility benefits in unadjusted models, although these associations became statistically insignificant after controlling for confounders.

Conclusions

Medical shoes effectively reduce pain and improve mobility in individuals with flatfoot, particularly in activities requiring joint flexibility and strength. However, their impact on long-term disability remains inconclusive. Integrating medical shoes with physiotherapy and behavioral strategies may further optimize outcomes. We recommend future longitudinal studies to evaluate sustained benefits and refine intervention strategies.

## Introduction

The foot, one of the most important organs in the body, balances the body's weight in addition to supporting it with its 33 joints, 26 bones, and more than 100 muscles [1]. Arcus transversalis, arcus longitudinalis pars medialis, and arcus longitudinalis pars lateralis are the three-foot arches. These arches expand and collapse when weight increases, then rebound as weight decreases [2]. Foot arches act as a shock absorber during movements, supporting body weight and propelling the body forward, and flexible arches let the foot conform to weight distribution and keep the surface pressed [3].

When the structural integrity of the arch is compromised, walking causes the ankle to tilt inward toward the body’s midline, increasing strain on the knees, hips, and lower back. This occurs as the body is unable to effectively transfer its weight to the anterior part of the foot. The degradation of the foot's arch structure can occur either before or after birth. People who frequently stand on uneven surfaces at work are at higher risk of developing pes planus [4]. Pes planus can also arise when the plantar fascia is overburdened, often due to factors such as being overweight, systemic illnesses, neurological disorders, or muscular imbalances [4,5].

Issues associated with pes planus have been shown to significantly impact individuals' quality of life and their ability to perform daily activities. These structural changes may lead to gait abnormalities and cause pain in the foot, calf, and back, interfering with activities like exercising, prolonged standing, and walking. This, in turn, affects physical fitness and overall quality of life. Abnormal foot arch deviations alter gait mechanics, resulting in excessive strain on bones and soft tissues, and these structural changes negatively impact functional efficiency. The resulting pain, reduced range of motion, and diminished muscular strength can trigger compensatory mechanisms that exacerbate the dysfunction, further impairing mobility and overall well-being [6].

Several studies have reported that the prevalence of pes planus ranges from 5% to 14% [7-9], and pes planus was found to be more prevalent among individuals with cardiovascular diseases, obesity, and diabetes, largely due to their sedentary lifestyle [8]. These conditions not only affect individuals' health but also negatively impact a nation’s economy and healthcare system. To alleviate pain and improve the quality of life for individuals with pes planus, conservative treatments are commonly preferred [10]. These include the use of insoles and orthotics, physiotherapy interventions, and, in some cases, surgical procedures. Foot orthotics, often placed inside shoes, are also used to mitigate the adverse effects of pes planus.

A systematic review of randomized control trials showed that custom-made orthoses significantly reduced pain intensity and disability in children with bilateral flat feet compared to supportive shoes alone. A second trial found no difference in foot pain between custom-made orthoses, prefabricated orthoses, and the control group. A third trial found no adverse effects, with no difference in the number of participants with foot pain between the three groups. No adverse effects were reported in all three trials [11]. These findings are inconclusive about the use of non-surgical interventions for pediatric pes planus, which underscores the need for more studies to explore conservative interventions.

In Saudi Arabia, there are currently no studies to evaluate the effectiveness of medical shoes in managing pain associated with flatfoot. Therefore, we conducted this study to address this gap in the literature and provide valuable insights into potential treatment options. This study aimed to assess the effectiveness of medical shoes in reducing mechanical pain and disability among patients with flatfoot (pes planus) in Saudi Arabia.

## Materials and methods

Study design and setting

This study employed a one-year retrospective cohort design to evaluate the impact of medical shoes on individuals with flatfoot. The research was conducted in Saudi Arabia and targeted individuals diagnosed with flatfoot. Two groups were identified: individuals who used medical shoes and those who did not.

Medical shoes were defined as specially designed footwear crafted to provide stability, arch support, and proper foot alignment for individuals with low or fallen arches. These shoes are engineered to address the unique needs of flatfooted individuals by preventing excessive inward rolling of the foot (overpronation), reducing foot pain, and improving overall gait. Constructed with supportive and cushioned materials, they enhance comfort and promote proper foot mechanics, ensuring optimal functionality and pain relief for those with flat feet.

Study population and sampling

The study population consisted of Saudi residents aged 18-65 years who were diagnosed with flatfoot or self-reported the condition. A two-stage sampling technique was employed: stage 1 involved the selection of healthcare and orthopedic facilities in Saudi Arabia as data sources to ensure geographical diversity and representation. Stage 2 entailed participant selection, where individuals meeting the inclusion criteria were identified from medical records, ensuring a balanced distribution of participants using and not using medical shoes.

Sample size determination

The sample size was calculated based on the prevalence of flatfoot in Saudi Arabia, estimated at 20% of the adult population [12]. Using a 95% confidence interval (CI) and a margin of error of ±5%, the required sample size was determined to be 400 participants. To account for potential dropouts or incomplete records, an additional 10% were recruited, resulting in a target sample size of 440 individuals.

Inclusion and exclusion criteria

The study included Saudi residents aged 18-65 years who were diagnosed with flatfoot by a healthcare professional and with a history of at least six months of either using or not using medical shoes. The exclusion criteria were as follows: individuals younger than 18 years or those older than 65 years, those with coexisting foot deformities such as clubfoot or severe bunions, those having incomplete or inaccessible medical records, or patients who had undergone surgical interventions for flatfoot correction during the study period.

Data collection

Data were collected retrospectively from medical records and included demographic characteristics such as age, weight, and height. Clinical data included the duration of flatfoot diagnosis, history of pain or discomfort, and associated comorbidities, including obesity and diabetes. Intervention-related data focused on the use of medical shoes, specifically their duration and frequency of use. Outcomes were assessed through validated tools, including pain severity, disability, and quality of life measures. The Foot Function Index (FFI) [13] was used to evaluate the severity of pain and functional limitations, capturing specific domains such as pain during standing, walking, and at the end of the day, as well as the worst pain experienced during the previous week. Disability measures addressed participants’ ability to perform activities such as walking various distances, climbing stairs, and standing on their toes. Long-term disability indicators, including the use of assistive devices and physical activity restrictions, were also documented.

Data analysis

Data were entered into Microsoft Excel and subsequently exported to SPSS Statistics version 29.0 (IBM Corp., Armonk, NY) for statistical analysis. Descriptive statistics, including minimum, maximum, mean, and standard deviation (SD), were computed for demographic variables such as age, weight, and height. Inferential statistics were applied to compare outcomes between participants using medical shoes and those who did not. The Mann-Whitney U test was used for non-parametric comparisons of pain and disability measures. Key outcomes, including pain severity and difficulty performing functional activities, were analyzed to identify statistically significant differences between the two groups.

Logistic regression analysis was conducted to evaluate the relationship between medical shoe use and key pain and disability measures. Logistic regression models were used to adjust for potential confounders, such as age, gender, and comorbid conditions, and to assess the independent effect of medical shoe usage on pain reduction and mobility improvement. Results were considered statistically significant at a p-value<0.05, and odd ratios (OR), as well as CIs, were calculated to measure the strengths of the identified associations.

Ethical consideration

Ethical approval was obtained from the Taif University Ethics Committee (no. 45-086). Informed consent was waived for retrospective data analysis, but anonymity and confidentiality of participants' information were maintained throughout the study.

## Results

Table 1 presents the demographic characteristics of the cohort. The mean age of participants was 25.07 years (SD: 7.99), ranging from 12 to 61 years. The average weight was 59.99 kg (SD: 14.88), and the average height was 162.3 cm (SD: 8.08).

**Table 1:** Demographic characteristics of the participants SD: standard deviation

Parameters	Minimum	Maximum	Mean	SD
Age (years)	12.00	61.00	25.07	7.99
Weight (kg)	34.00	160.00	59.99	14.88
Height (cm)	144.0	189.0	162.3	8.08

Table 2 compares self-reported pain levels between participants who used medical shoes and those who did not. The Mann-Whitney U test revealed that patients using medical shoes demonstrated significantly lower pain when standing (p=0.024), with significantly reduced end-of-day pain (p=0.010) and pain severity (p=0.001). Although morning first-step pain and walking pain did not reach statistical significance, the observed trends suggested improvements among medical shoe users compared to non-users. 

**Table 2:** Impact of medical shoe use on self-reported foot pain over the past week *Statistically significant SD: standard deviation

Items	Using medical shoes						
	No (n=202)			Yes (n=198)			P-value
	Mean	SD	Mean rank	Mean	SD	Mean rank	
In the morning, when you take your first step	0.748	1.75	195.6	1.071	2.15	205.5	0.281
When walking?	1.450	1.99	209.8	1.328	2.27	191.0	0.074
When standing?	1.470	2.21	211.8	1.111	2.06	189.0	0.024*
How is your pain at the end of the day?	2.946	2.96	214.8	2.247	2.81	185.9	0.010*
How severe was your pain at its worst?	3.748	3.21	219.5	2.753	3.25	181.1	0.001*

Table 3 compares self-reported difficulty levels (disability) between participants using and not using medical shoes for various activities (e.g., walking, climbing stairs, standing on toes). The Mann-Whitney U test showed that participants wearing medical shoes demonstrated significantly reduced difficulty in three specific domains: walking four blocks (p=0.012), climbing stairs (p=0.014), and standing on toes (p=0.007). However, medical shoe use did not have a statistically significant impact on most activity-related disability measures (all p>0.05)

**Table 3:** Impact of medical shoe use on self-reported difficulty in performing activities (disability scale) *Statistically significant SD: standard deviation

Items	Using medical shoes						
	No (n=202)			Yes (n=198)			P-value
	Mean	SD	Mean rank	Mean	SD	Mean rank	
When walking in the house?	0.812	1.55	200.7	1.035	2.02	200.3	0.965
When walking outside the house?	1.733	2.25	206.0	1.687	2.59	194.9	0.300
When you walk four blocks?	2.351	2.63	214.2	1.793	2.54	186.5	0.012*
When climbing stairs?	2.010	2.59	213.5	1.636	2.61	187.3	0.014*
When going downstairs?	1.163	1.87	205.8	1.202	2.18	195.1	0.293
When standing on your toes?	1.832	2.58	214.4	1.288	2.27	186.3	0.007*
When standing up from a chair?	1.109	2.05	201.8	1.051	1.96	199.2	0.790
When climbing the sidewalk?	1.089	1.98	202.8	1.182	2.16	198.2	0.644
When running or walking quickly?	2.347	2.67	207.7	2.172	2.87	193.2	0.190

As shown in Table 4, the Mann-Whitney U test was employed to examine long-term disability indicators among patients using medical shoes. The results revealed subtle but not statistically significant differences across assistive device usage and physical activity restrictions (all p>0.05).

**Table 4:** Impact of medical shoe use on long-term disability indicators SD: standard deviation

Items	Using medical shoes						
	No (n=202)			Yes (n=198)			P-value
	Mean	SD	Mean rank	Mean	SD	Mean rank	
Do you use an assistive device (cane, walker, crutches, etc.) inside the home?	0.292	1.25	197.3	0.429	1.56	203.7	0.275
Do you use an assistive device (cane, walker, crutches, etc.) outside the home?	0.312	1.41	196.7	0.551	1.83	204.4	0.195
Do you restrict physical activities?	1.475	2.39	208.6	1.247	2.38	192.3	0.102

Finally, the logistic regression analysis explored the relationship between various pain and disability measures and medical shoe use (Table 5). In the crude OR analysis, several factors showed statistically significant associations with medical shoe use, including end-of-day pain (OR: 1.088, 95% CI [1.015-1.166], p=0.017), worst pain severity (OR: 1.10, 95% CI [1.034-1.170], p=0.002), walking four blocks (OR: 1.088, 95% CI [1.007-1.176], p=0.033), and standing on toes (OR: 1.098, 95% CI [1.010-1.193], p=0.028). However, after adjusting for potential confounding factors, these associations largely attenuated, with none of the variables maintaining statistical significance in the adjusted model, suggesting that these factors may not be independent predictors of medical shoe use when controlling for other variables. 

**Table 5:** Logistic regression analysis predicting medical shoe use from foot pain and disability measures *Statistically significant aOR: adjusted odds ratio; CI: confidence interval; OR: odds ratio

Predictors	Crude odds ratio		Adjusted odds ratio	
	OR [95% CI]	P-value	aOR [95% CI]	P-value
When standing?	1.083 [0.986-1.189]	0.095	NA	NA
How is your pain at the end of the day?	1.088 [1.015-1.166]	0.017*	0.966 [0.840-1.112]	0.633
How severe was your pain at its worst?	1.10 [1.034-1.170]	0.002*	1.116 [0.988-1.259]	0.077
When you walk four blocks?	1.088 [1.007-1.176]	0.033*	0.990 [0.878-1.116]	0.872
When climbing stairs?	1.057 [0.980-1.141]	0.152	NA	NA
When standing on your toes?	1.098 [1.010-1.193]	0.028*	1.037 [0.917-1.173]	0.563

## Discussion

The results of this study revealed significant pain reduction and improved mobility in individuals using medical shoes compared to those who did not. These findings align with previous research that highlights the potential benefits of medical shoes in managing pain and enhancing functional performance in flatfoot patients. However, the study also highlighted limitations in the impact of medical shoes on long-term disability indicators, suggesting the need for further investigation and refinement of interventions.

The significant reduction in pain levels seen in this study, particularly in domains such as standing, end-of-day pain, and worst pain severity, supports findings from previous studies conducted outside Saudi Arabia reporting that custom-made orthotics and medical footwear provide considerable relief from pain and discomfort in individuals with flatfoot [14,15]. It was established that orthotic interventions reduce pain intensity by improving foot posture [16]. Notably, the pronounced improvement in worst pain severity among medical shoe users suggests that these interventions are effective for managing severe cases of flatfoot-related pain. However, the lack of statistically significant improvement in walking and morning first-step pain, despite observable trends, differs from the findings of previous studies, which reported consistent pain reduction across all domains with custom orthotics [14,17,18]. This discrepancy may be attributed to differences in the population studied, footwear design, or treatment adherence.

Functional improvements in activities such as walking four blocks, climbing stairs, and standing on toes confirm evidence that medical footwears enhance biomechanical efficiency and reduce the strain on lower extremities [15,16,18]. The improvements noted in this study emphasize the potential of medical shoes to support activities requiring greater joint flexibility and strength. However, the lack of significant improvement in other activities, such as walking outside the house or running, agrees with studies suggesting that while medical shoes may enhance stability and strength, their impact on high-impact activities remains limited [19].

Interestingly, the study found no significant difference in long-term disability indicators, such as the use of assistive devices or physical activity restrictions, which contrasts with research by Guldemond et al., which suggested that sustained use of orthotic devices can reduce dependency on assistive tools over time [20]. The disparity may stem from the shorter observation period of this study or differences in the severity of flatfoot among participants. Interestingly, it was suggested that, when engaging in activities outside of the home, orthotic users may benefit the best from using assistive technology and orthotic devices simultaneously [21].

The results underscore the potential of medical shoes as a conservative intervention for managing flatfoot-related pain and disability. However, the findings also suggest that their impact may be more pronounced in alleviating pain and improving specific functional tasks rather than addressing broader disability measures. These insights highlight the need for a multi-faceted approach to flatfoot management. Integration of physiotherapy by combining medical shoe use with targeted physiotherapy programs could enhance outcomes. Previous research demonstrated that strengthening and stretching exercises significantly improve foot posture and reduce pain in flatfoot patients [22,23]. Future studies could evaluate the synergistic effects of such combined interventions. This study's results showed variability in pain relief and functional improvement, suggesting that customized medical shoes tailored to individual biomechanical needs might lead to better outcomes.

Evidence from previous studies also supports the use of customized footwear in improving patient satisfaction and therapeutic effectiveness [17,24]. Since adherence to medical shoe use can influence outcomes, integrating behavioral strategies to improve compliance may be beneficial. Ryan et al. [25] have shown that motivational interviewing and patient education enhance adherence to prescribed interventions. Thus, educating patients on the benefits of using medical shoes and proper foot care can enhance compliance and outcomes. Patients should be informed about the potential improvements in pain and disability associated with the consistent use of medical shoes. Emerging technologies, such as pressure-sensing insoles and biomechanical feedback systems, could be integrated into medical shoes to optimize their therapeutic potential [26]. Research has indicated that biofeedback systems are effective in improving gait mechanics [26,27], which could complement the benefits of medical footwear. The lack of significant findings in long-term disability indicators calls for longitudinal studies to assess the sustained impact of medical shoes. Extending the follow-up period could clarify their role in reducing assistive device dependency and improving physical activity levels.

We did not find any statistically significant independent predictor of using medical shoes, though end-of-day pain, worst pain severity, walking four blocks, and standing on toes were significant in the univariate logistic analysis. After adjusting for confounders, these predictors became statistically insignificant, suggesting that these factors may not be independent predictors of medical shoe use when controlling for other variables. These findings indicate that the decision to use medical shoes is likely influenced by a complex interplay of factors beyond simple pain and disability measures. Therefore, future extensive longitudinal studies are recommended to identify predictors of using medical shoes among individuals with pes planus to inform targeted interventions to improve adherence, healthcare decision-making and recommendations, and correct use.

This study has a few limitations. The retrospective design and reliance on self-reported data may introduce bias and limit the generalizability of the findings. Additionally, the lack of information on adherence to medical shoe use and variability in shoe designs may affect the observed outcomes. Future research should address these limitations by employing prospective designs, standardizing footwear interventions, and incorporating objective measures such as gait analysis and biomechanical assessments.

## Conclusions

The findings show that medical shoes significantly reduce mechanical pain and disability in patients with flat feet compared to non-medical shoes. Participants who used medical shoes reported lower pain levels and improved functionality in daily activities compared to those who did not. This study adds to the growing body of evidence supporting the use of medical shoes for managing flatfoot-related pain and functional limitations. However, while the findings highlight their efficacy in reducing pain and enhancing mobility in specific domains, their impact on long-term disability indicators remains inconclusive. By integrating customized footwear, physiotherapy, and behavioral strategies, future interventions could further optimize outcomes for flatfoot patients. Moreover, exploring innovative technologies and conducting longitudinal studies will help address current gaps in the literature, ultimately advancing the care and quality of life for individuals with flatfoot.

## References

[1] Alazzawi S, Sukeik M, King D, Vemulapalli K (2017). Foot and ankle history and clinical examination: a guide to everyday practice. World J Orthop.

[2] Gwani AS, Asari MA, Mohd Ismail ZI (2017). How the three arches of the foot intercorrelate. Folia Morphol (Warsz).

[3] Kirby KA (2017). Longitudinal arch load-sharing system of the foot. Revista Española de Podología.

[4] Michaudet C, Edenfield KM, Nicolette GW, Carek PJ (2018). Foot and ankle conditions: pes planus. FP Essent.

[5] Hashimoto T, Sakuraba K (2014). Strength training for the intrinsic flexor muscles of the foot: effects on muscle strength, the foot arch, and dynamic parameters before and after the training. J Phys Ther Sci.

[6] Bac A, Kaczor S, Pasiut S, Ścisłowska-Czarnecka A, Jankowicz-Szymańska A, Filar-Mierzwa K (2022). The influence of myofascial release on pain and selected indicators of flat foot in adults: a controlled randomized trial. Sci Rep.

[7] Lee MS, Vanore JV, Thomas JL (2005). Diagnosis and treatment of adult flatfoot. J Foot Ankle Surg.

[8] Stolzman S, Irby MB, Callahan AB, Skelton JA (2015). Pes planus and paediatric obesity: a systematic review of the literature. Clin Obes.

[9] Aenumulapalli A, Kulkarni MM, Gandotra AR (2017). Prevalence of flexible flat foot in adults: a cross-sectional study. J Clin Diagn Res.

[10] Park CH, Chang MC (2019). Forefoot disorders and conservative treatment. Yeungnam Univ J Med.

[11] Rome K, Ashford RL, Evans A (2010). Non-surgical interventions for paediatric pes planus. Cochrane Database Syst Rev.

[12] Almansouf AS, Alosaimi MI, Alsinan SH, Almanea RK, Almansoof AS, Jawadi AH (2022). The prevalence of flatfoot among Saudi population: a systematic review. J Musculoskelet Surg Res.

[13] Budiman-Mak E, Conrad KJ, Roach KE (1991). The foot function index: a measure of foot pain and disability. J Clin Epidemiol.

[14] Bednarczyk E, Sikora S, Kossobudzka-Górska A, Jankowski K, Hernandez-Rodriguez Y (2024). Understanding flat feet: an in-depth analysis of orthotic solutions. J Orthop Case Rep.

[15] Mohaddis M, Maqsood SA, Ago E, Singh S, Naim Z, Prasad S (2023). Enhancing functional rehabilitation through orthotic interventions for foot and ankle conditions: a narrative review. Cureus.

[16] Simonsen MB, Næsborg-Andersen K, Leutscher PD, Hørslev-Petersen K, Woodburn J, Andersen MS, Hirata RP (2022). The effect of foot orthoses on gait biomechanics and pain among people with rheumatoid arthritis: a quasi-experimental study. Gait Posture.

[17] Lee HJ, Lim KB, Yoo J, Yoon SW, Yun HJ, Jeong TH (2015). Effect of custom-molded foot orthoses on foot pain and balance in children with symptomatic flexible flat feet. Ann Rehabil Med.

[18] Banwell HA, Mackintosh S, Thewlis D (2014). Foot orthoses for adults with flexible pes planus: a systematic review. J Foot Ankle Res.

[19] Orr R, Maupin D, Palmer R, Canetti EF, Simas V, Schram B (2022). The impact of footwear on occupational task performance and musculoskeletal injury risk: a scoping review to inform tactical footwear. Int J Environ Res Public Health.

[20] Guldemond NA, Leffers P, Sanders AP, Schaper NC, Nieman F, Walenkamp GH (2007). Daily-life activities and in-shoe forefoot plantar pressure in patients with diabetes. Diabetes Res Clin Pract.

[21] Stevens PM, Hafner BJ, Weber EL, Morgan SJ, Bamer AM, Salem R, Balkman GS (2024). Utilization of orthoses and assistive devices among a national sample of lower limb orthosis users. J Rehabil Assist Technol Eng.

[22] Kolhe PD, Sharath HV, Rathi SG, Patil DS (2024). Effect of foot rehabilitation exercises for painful flat foot in a 20-year-old female: a case study analysis. Cureus.

[23] Brijwasi T, Borkar P (2023). A comprehensive exercise program improves foot alignment in people with flexible flat foot: a randomised trial. J Physiother.

[24] Jannink M, van Dijk H, Ijzerman M, Groothuis-Oudshoorn K, Groothoff J, Lankhurst G (2006). Effectiveness of custom-made orthopaedic shoes in the reduction of foot pain and pressure in patients with degenerative disorders of the foot. Foot Ankle Int.

[25] Ryan CG, Grant PM, Tigbe WW, Granat MH (2006). The validity and reliability of a novel activity monitor as a measure of walking. Br J Sports Med.

[26] Jafarnezhadgero AA, Sorkhe E, Oliveira AS (2019). Motion-control shoes help maintaining low loading rate levels during fatiguing running in pronated female runners. Gait Posture.

[27] Cheung RT, Wong MY, Ng GY (2011). Effects of motion control footwear on running: a systematic review. J Sports Sci.

